# Caregiver Contribution to Self-Care of Chronic Illness Inventory: Evaluation of Measurement Properties in a Middle-Income Country

**DOI:** 10.3390/nursrep15020042

**Published:** 2025-01-26

**Authors:** Sajmira Adëraj, Alta Arapi, Rocco Mazzotta, Alessandro Stievano, Dasilva Taci, Dhurata Ivziku, Vicente Bernalte-Martí, Ercole Vellone, Gennaro Rocco, Maddalena De Maria

**Affiliations:** 1 Department of Biomedicine and Prevention, University of Rome Tor Vergata, 00133 Rome, Italy; sajmira.aderaj@students.uniroma2.eu (S.A.); alta.arapi@students.uniroma2.eu (A.A.); dasilva.taci@students.uniroma2.eu (D.T.); ercole.vellone@uniroma2.it (E.V.); 2 Department of Clinical and Experimental Medicine, University of Messina, 98122 Messina, Italy; alessandro.stievano@unime.it; 3 Center of Excellence for Nursing Culture and Research, Order of Nursing Professions of Rome, 00136 Rome, Italy; genna.rocco@gmail.com; 4 Department of Health Professions, Fondazione Policlinico Universitario Campus Bio-Medico, 00128 Rome, Italy; d.ivziku@policlinicocampus.it; 5 Department of Nursing, Faculty of Health Sciences, University of Jaume I, 12071 Castellón, Spain; bernalte@uji.es; 6 Faculty of Nursing and Midwifery, Wroclaw Medical University, 50-996 Wrocław, Poland; 7 International Center for Nursing Research Montianum Our Lady of Good Counsel, Catholic University, 1000 Tirana, Albania; 8 Department of Life Science, Health, and Health Professions, Link Campus University, 00165 Rome, Italy; m.demaria@unilink.it

**Keywords:** caregivers, self-care, chronic illness, older adults, instrument, measurement properties testing, validity, reliability

## Abstract

**Background/Objectives:** Caregivers engage in essential tasks that support patients’ well-being and survival, including administering medications, promoting healthy lifestyle choices, and monitoring and managing symptoms. To date, no valid and reliable instrument is available to assess Caregiver Contribution (CC) to self-care in chronic conditions in middle-income countries such as Albania. **Aim:** To evaluate the measurement properties (structural and construct validity, internal consistency reliability, stability, and measurement error) of the instrument CC to Self-Care of the Chronic Illness Inventory (CC-SC-CII) in Albanian caregivers caring for elderly people affected by multiple chronic conditions. **Methods:** A cross-sectional study. We enrolled caregivers of patients with multiple chronic conditions, from August 2020 to April 2021, if they were (a) 18 years of age or older and (b) were identified by the patient as the main unpaid caregiver. **Results**: Confirmatory factor analysis confirmed the two-factor structure of the CC to Self-Care Maintenance and Management scales and the one-factor structure of the CC to Self-Care Monitoring scale. Reliability estimates were adequate for all (coefficients ranging between 0.827 and 0.961). The construct’s validity was supported. The measurement error was adequate. **Conclusions:** The Albanian version of the CC to Self-Care of the Chronic Illness Inventory features sound measurement properties and is a valid and reliable instrument for assessing caregiver contribution to patient self-care behaviors in the Albanian population.

## 1. Introduction

The prevalence of chronic diseases is increasing worldwide, including low/middle-income countries (LMICs). Chronic diseases kill 41 million people each year, equivalent to 71% of all deaths globally [1]. Seventy-seven percent of all chronic disease deaths occur in LMICs [1]. In LMICs, such as Albania [2] about 85% of the overall burden of healthcare and about 94% of proportional mortality is attributable to chronic diseases [3].

Chronic diseases disproportionately affect older adults and can lead to increased disability, mortality, and long-term care costs [4,5]. The WHO-consolidated guidelines support and promote self-care interventions for every country and economic setting as critical components to strengthen primary healthcare and counteract the burden of chronic illness [6,7]. According to the Middle Range Theory of Self-Care of Chronic Illness, self-care is defined as “a process of maintaining health by promoting health and managing illness” [8]. It comprises the following three different dimensions: self-care maintenance (behaviors aimed at maintaining the stability of chronic illnesses), self-care monitoring (behaviors aimed at monitoring and recognizing signs and symptoms), and self-care management (behaviors of managing signs and symptoms when they occur). Although there is a growing body of literature regarding the benefits of self-care [6,9], there is also the literature demonstrating that self-care is complex for patients to implement, especially older adults, due to symptom overlap, low self-efficacy, and sensory and perceptual alterations [10]. In these cases, the presence of informal caregivers (hereinafter referred to as caregivers), defined as a family member or a significant other providing unpaid help [11], is crucial to support patients with chronic conditions in performing self-care [12,13].

The support of caregivers is conceptualized as Caregiver Contribution (CC) to self-care, defined as “a process of recommendations for (or substitutions for) the care-recipient in performing behaviors that help maintain the chronic illness’s stability and manage signs and symptoms” [11]. Caregivers engage in essential tasks that support patients’ well-being and survival, including administering medications, promoting healthy lifestyle choices, and monitoring and managing symptoms, etc. Such caregiver contributions affect patients’ outcomes, resulting in physical and psychological improvements [14]. Additionally, emotional support provided by caregivers decreases emergency room visits and mitigates worsening symptoms [15]. For these reasons, CC to self-care can be crucial for improving patient clinical outcomes.

A widely used and theoretically grounded instrument for measuring caregiver contribution to self-care in chronically ill patients is the Caregiver Contribution to Self-Care of Chronic Illness Inventory (CC-SC-CII). It reflects the Self-Care of Chronic Illness Inventory (SC-CII) used for patients and explores the extent to which informal caregivers recommend that patients perform self-care [16,17]. The CC-SC-CII measures the CC to self-care process consisting of three theoretical dimensions—CC to self-care maintenance, CC to self-care monitoring, and CC to self-care management. The CC-SC-CII has been translated into several languages, such as Arabic, Chinese, Italian, Portuguese, and Thai, but not Albanian. In previous studies [18,19,20] the CC-SC-CII has been proven to be a sound instrument with good measurement properties and with good factorial and concurrent validity and internal consistency. However, no previous studies have tested the measurement properties of the CC-SC-CII in LMICs. Additionally, no previous studies have tested the stability and measurement error of the CC-SC-CII.

Currently, there is no validated and reliable instrument to evaluate CC to self-care in chronic conditions in a LMIC like Albania. As a result, it is not possible to adequately assess CC to self-care behaviors. This study evaluated the measurement properties (structural and construct validity, internal consistency reliability, stability, and measurement error) of the CC-SC-CII in Albanian caregivers caring for older adults affected by multiple chronic conditions (MCCs).

## 2. Materials and Methods

### 2.1. Design

For this study, we used baseline data from the ongoing Albanian longitudinal study whose objective is to describe patients’ self-care and caregiver contribution to patients’ self-care in the context of MCCs.

### 2.2. Participants and Setting

A convenience sample of patient–caregiver dyads in outpatient and community settings was recruited in all regions of Albania. Inclusion criteria for patients were age ≥ 65 years, diagnosed with diabetes mellitus (DM), chronic obstructive pulmonary disease (COPD), or heart failure (HF), and at least one other chronic condition. Inclusion criteria for caregivers were age ≥ 18 years and being identified as the primary caregiver by their care recipients. Patients with dementia and/or cancer were excluded. Both members of each dyad were excluded if either one of them was not eligible.

### 2.3. Data Collection

Data collection was carried out through face-to-face interviews, 30 min on average, between August 2020 and April 2021. The interviews were conducted by nurse research assistants. They underwent a training program provided by the research team, which included sessions on interview techniques, the use of data collection tools, and strategies to minimize bias. Additionally, mock interviews were conducted, followed by feedback sessions to ensure consistency and reliability in data collection. Once potential participants were identified, the objectives of the study were explained and informed consent was obtained.

### 2.4. Measurements

The patient’s self-care was measured by the Albanian version of the Self-Care of Chronic Illness Inventory (SC-CII-Al) [21]. It is a theoretically grounded instrument composed of 19 items grouped in the following three scales: self-care maintenance (7 items), self-care monitoring (5 items), and self-care management (7 items). The SC-CII-Al uses a 5-point Likert scale that ranges from 1 (Never) to 5 (Always). Higher scores indicate improved self-care, with three different 0–100 standardized scores computed for each SC-CII-Al scale. Only patients who experienced signs and symptoms related to their chronic illnesses could complete the self-care management scale. The SC-CII-Al was validated by a previous study [21].

The Albanian version of the Caregiver Contribution to Self-Care of Chronic Illness Inventory (CC-SC-CII-Al) was used to measure the CC to patient’s self-care. This instrument was developed based on the same items as the Self-Care of Chronic Illness Inventory, whose measurement properties have been well-supported. The CC-SC-CII-Al is composed of three separate scales that measure the theoretical dimensions of the CC to self-care process as follows: CC to self-care maintenance (7 items), CC to self-care monitoring (5 items), and CC to self-care management (6 items). The CC to Self-Care Maintenance scale captures the caregiver’s contribution to behaviors aimed at maintaining the physical and mental stability of a chronic illness (i.e., how often do you recommend that the person for whom you care eat a special diet?). The CC to Self-Care Monitoring scale measures the caregiver’s contribution to behaviors aimed at monitoring the signs and symptoms of the care recipient’s chronic illness (i.e., How often do you monitor the condition of the person for whom you care?). The CC to Self-Care Management scale evaluates the caregiver’s contribution to responding to symptoms of chronic illness exacerbation (i.e., how likely are you to recommend the person for whom you care tell his/her healthcare provider about the symptoms at the next office visit?). Consistent with previous research [21] on the SC-CII, item #7 (avoid tobacco smoking), which describes CC’s efforts to encourage avoiding tobacco smoking, and item #14 (How quickly did you recognize the symptoms of his/her illness?), which relates to CC’s symptom recognition, were excluded from the analyses. The CC-SC-CII-Al uses a five-point Likert scale to record responses from 1 (never) to 5 (always) for the CC to Self-Care Maintenance and CC to Self-Care Monitoring scales, and from 1 (not likely) to 5 (very likely) for the CC to Self-Care Management scale. All caregivers can complete the Self-Care Maintenance and Self-Care Monitoring scales. Caregivers caring for asymptomatic patients do not complete the Self-Care Management scale [20]. Each scale provides a 0–100 standardized score, where higher scores indicate a higher contribution to self-care. The instrument is available at https://self-care-measures.com (accessed on 10 August 2024). The translation of the original English version to Albanian followed the Principles of Good Practice for the translation, cultural adaptation, and linguistic validation of clinician-reported outcomes, observer-reported outcomes, and performance outcome measures [22]. Specifically, this process began with two bilingual translators independently translating the instrument from English to Albanian. A third translator then reviewed and reconciled the translations in collaboration with the research team. Next, two additional translators back-translated the reconciled version into English to ensure accuracy. The research team reviewed and refined this version, addressing ambiguities with the translators, and obtained approval from the authors of the CC-SC-CII.

The Positive Aspect of Caregiving scale (PACs-9) was used to measure the positive feelings resulting from care provision among family caregivers [23]. The PACs-9 is a self-reported multidimensional tool that assesses positive feelings resulting from care provision among family caregivers, exploring aspects such as Self-affirming and Life-enriching. The PACs-9 comprises 9 items requiring a five-point Likert-type scale response from 1 (disagree a lot) to 5 (agree a lot). The overall PACs score, comprising all nine items, ranges from 9 to 45 where a higher score reflects a more positive perception of the caregiving experience [23].

Socio-demographic variables (i.e., age, gender, marital status, level of education, employment, income, cohabitation of the caregiver and care recipient, the existence of a secondary caregiver, and the number of hours per week) were collected by a structured questionnaire developed ad-hoc for the study.

### 2.5. Ethical Considerations

The Albanian SODALITY study was approved by the Ethics Committee of the Catholic University of Our Lady of Good Counsel in Tirana. Potential study participants were fully informed about the study aims and reassured that their data would be kept confidential. All participants provided verbal and written informed consent before participation. The study was conducted in accordance with the principles of the Declaration of Helsinki and in compliance with current legislation on clinical trials.

### 2.6. Data Analysis

Missing data were assessed at the item level. Descriptive statistics (frequency, percentage, mean, and standard deviation [SD] coefficients, where appropriate) were computed to describe the sample characteristics and the CC-SCCII-Al items. The normality of the CC-SCII-Al items, kurtosis, and skewness were evaluated [24]. Consistent with the recent literature, we started the analysis by testing the scale dimensionality and subsequently the reliability [25].

To test the structural validity or dimensionality of the scale, confirmatory factor analysis (CFA) was performed. Due to the non-normal distribution of SC-CII-Al items, the maximum likelihood robust (MLR) estimator [26] was used for parameter estimation. According to previous validation studies conducted on self-care instruments [18,19,20], three individual CFAs, one per each scale (CC Self-Care Maintenance, CC Self-Care Monitoring, and CC Self-Care Management) were tested. For CC-Self-Care Maintenance and CC-Self-Care Monitoring scales, the structural model identified in a previous study [20] was tested. For the CC-Self-Care Management scale, we tested the structure of the Self-Care Management scale (patient scale version) presented in a recent study [21]. Specifically, we tested, for CC Self-Care Maintenance, a two-factor model with CC health promoting behaviors and illness-related factors (measured by items #1, #3, #8, and #2, #4, #5, #6, respectively); for the CC-Self-Care Monitoring scale, a unidimensional model (measured by items #9–13); and finally, for the CC-Self-Care Management scale, a two-factor model with autonomous behaviors and consulting behavior factors (measured by items #15, 16, 17, 20, and #18–19, respectively). Additionally, for the CC-Self-Care Maintenance and CC-Self-Care Management scales, a second-order model was tested considering the correlation between factors. Model fit was examined using the following fit indices: χ^2^ statistics, comparative fit index (CFI), Tucker and Lewis index (TLI), Root mean square error of approximation (RMSEA), and Standardized root mean square residual (SRMR) [27,28]. The goodness of fit values was interpreted following the literature recommendations [29,30].

The construct validity of the CC-SC-CII-Al was evaluated via hypothesis testing by investigating the correlations among scales. The hypotheses were tested using the Pearson correlation coefficient. A correlation coefficient ranging between 0.10 and 0.29 was considered weak, a coefficient ranging between 0.30 and 0.50 was considered moderate, and a coefficient > 0.50 was considered strong [31]. Several hypotheses were posed. The hypothesis included the following: 1. Scores of each scale composing CC-SC-CII-Al would positively correlate with the corresponding scales of the SC-CII-Al, as found in previous studies [20]. 2. Positive correlations among the scores of PACs, Self-affirming and Life-enriching factors, with each CC-SC-CII-Al scale. The rationale of this hypothesis is that caregiver contribution to self-care is a fulfilling endeavor for family caregivers [32]. Internal consistency reliability was computed by estimating the composite reliability coefficient for each factor of the CC-Self-Care Maintenance and CC-Self-Care Management scales [33]. Cronbach’s alpha coefficient, which assumes that the items reflect a unidimensional structure, was computed for the CC-Self-Care Monitoring scale. The global reliability index for multidimensional scales [34], a more appropriate reliability coefficient which considers the scale’s multidimensionality, was also computed to test the reliability of the overall CC Self-Care Maintenance and the CC Self-Care Management scales. Values ≥ 0.70 were considered adequate [35].

To evaluate the stability of the CC-SCCII-Al, the test–retest reliability was assessed by administering the instrument within two weeks to a subset of 50 patients with stable multiple chronic conditions. Intra-class correlation coefficients (ICCs) through a two-way random effects model were calculated for the scores of each scale. An ICC ≥ 0.75 is considered good reliability and ≥ 0.90 is considered excellent reliability [36].

To evaluate responsiveness to changes, a measure of instrument precision, we tested the CC-SC-CII scale measurement error by computing the standard error of measurement (SEM) and the smallest detectable change (SDC). SEM was computed using the following formula: standard deviation (SD) × √ (1 − reliability coefficient) [37], where the SD was the SD of the CC-SC-CII scale score, and the reliability coefficients were the Cronbach’s alpha and the multidimensional model-based reliability for unidimensional and bidimensional scales, respectively. The smaller the SEM and the SDC, the more precise the instrument.

Statistical analyses were performed using SPSS Version 26, except for CFAs, which were performed using Mplus Version 8.4 [38].

## 3. Results

### 3.1. Characteristics of the Sample

Table 1 shows the characteristics of the total sample (n = 250). In brief, caregivers had a mean age of 49.9 (±15.6) years, were mostly female (66.4%), married (82.8%), with a low level of education (96.8%), and were the patients’ children (38.4%) or spouses/partners (31.2%).

### 3.2. Item Descriptive Analysis

Detailed item descriptive characteristics are presented in Table 2. The mean score of each item ranged from 3.51 to 4.74. The lowest mean scores (meaning lower self-care) were found for item #3 (3.99 ± 1.05), #11 (4.13 ± 0.88), and #20 (3.51 ± 1.16) for the Self-Care Maintenance, Self-Care Monitoring, and Self-Care Management scales, respectively.

The highest means scores (meaning higher self-care) were found for items #5 (4.57 ± 0.71), #9 (4.50 ± 0.67), and #18 (4.53 ± 0.71) for the Self-Care Maintenance, Self-Care Monitoring, and Self-Care Management scales, respectively.

Several items had skewness and/or kurtosis indices > |1| showing the non-normality of item distribution. Regarding item #14, 94.4% of caregivers reported recognizing the symptoms and 28% of them somewhat quickly.

### 3.3. Testing the Structural Validity of the CC-SC-CII Albanian Version

#### 3.3.1. Caregiver Contribution to Self-Care Maintenance Scale

**Figure 1:**
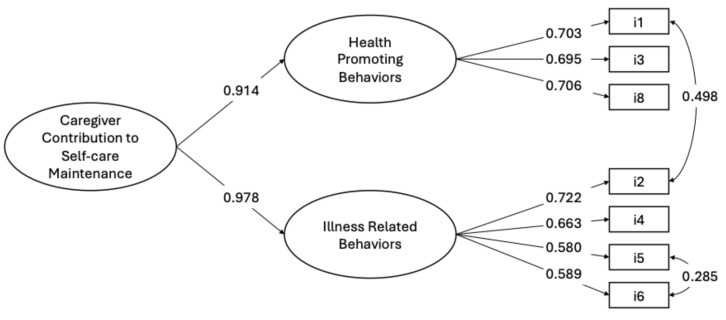
Confirmatory factor analysis of CC to Self-Care Maintenance scale (N = 249 caregivers). Note. The results are derived from Mplus fully standardized solutions, with all coefficients reaching statistical significance (*p* < 0.05). The values shown next to the single-headed arrows represent factor loadings, while the values next to the double-headed arrows indicate correlation coefficients.

The two-factor model with CC Health Promoting Behaviors and CC illness-related factors was tested. The goodness-of-fit indices of this model were not good, as shown by the following metrics: χ^2^ (13, N = 249) = 54.822, *p* < 0.001, CFI = 0.894, TLI = 0.829, RMSEA = 0.114 (90% CI = 0.084 0.146), *p* < 0.001, SRMR = 0.056. An inspection of modification indicates two pairs of items as follows: #1 and #2, and #5 and #6 showed an excessive residual covariance. The closeness of these items in the scale may have increased the shared covariance, an effect described by Weijters and colleagues [39] as the *proximity effect*. According to Bagozzi [35] and Fornel [40], we specified these covariances and the fit model improved considerably as follows: χ^2^ (11, N = 250) = 19.369, *p* = 0.055, CFI = 0.976, TLI = 0.960, RMSEA = 0.055 (90% CI = 0.000 0.095), *p* = 0.370, SRMR = 0.035. All factor loadings were significant and greater than 0.580. Since the two factors were significantly correlated (r = 0.895, *p* < 0.001), a second-order model was specified to yield the same fit indices as those of the first order, indicating that the Self-Care Maintenance scale shows a two-factor structure at the first-order level and is unidimensional at the second-order level (Figure 1).

#### 3.3.2. Caregiver Contribution to Self-Care Monitoring Scale

**Figure 2:**
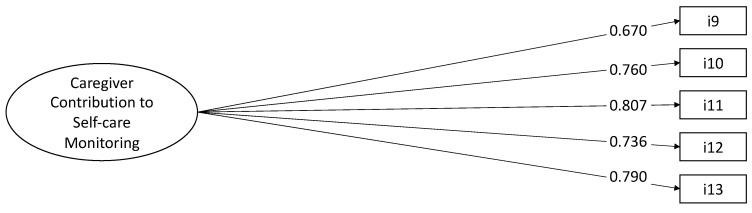
Confirmatory factor analysis of CC to Self-Care Monitoring scale (N = 250 caregivers). Note. The results are derived from Mplus fully standardized solutions, with all coefficients reaching statistical significance (*p* < 0.05). The values shown next to the single-headed arrows represent factor loadings, while the values next to the double-headed arrows indicate correlation coefficients.

We posited that a single factor would underlie the five items composing the CC-Self-Care Monitoring scale, so we specified a one-factor model CFA, which showed good fit indices as follows: χ^2^ (5, N = 250) = 19.369, *p* = 0.069, CFI = 0.972, TLI = 0.945, RMSEA = 0.091 (90% CI = 0.000 0.172), *p* = 0.165, SRMR = 0.030. All factor loading were significant and greater than 0.670 (Figure 2).

#### 3.3.3. Caregiver Contribution to Self-Care Management Scale

We tested the two-factor model with autonomous behavior and consulting behavior factors which yielded the following unsatisfactory fit indices: χ^2^ (8, N = 236) = 62.216, *p* < 0.001, CFI = 0.800, TLI = 0.625, RMSEA = 0.169 (90% CI = 0.132 0.210), *p* = 0.370, SRMR = 0.061. The modification indices revealed that the poor fit was caused by excessive covariance between items #17 and #18, and #19 and #20.

**Figure 3:**
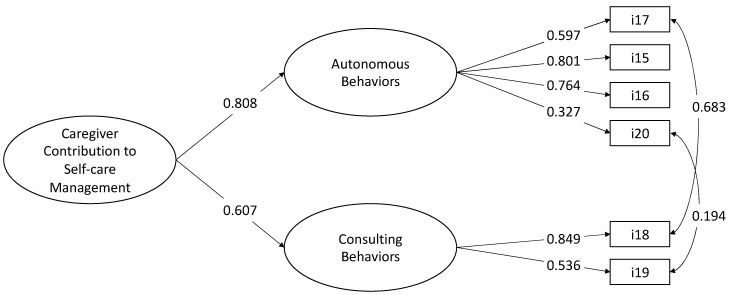
Confirmatory factor Management scale (N = 236 caregivers). Note. The results are derived from Mplus fully standardized solutions, with all coefficients reaching statistical significance (*p* < 0.05). The values shown next to the single-headed arrows represent factor loadings, while the values next to the double-headed arrows indicate correlation coefficients.

As for the CC to Self-Care Maintenance scale, the proximity effects might have increased the residual covariances between these items. We reran the model to allow the residuals of these items to correlate and the fit improved as follows: χ^2^ (6, N = 236) = 9.338, *p* = 0.1554, CFI = 0.988, TLI = 0.969, RMSEA = 0.049 (90% CI = 0.000 0.106), *p* = 0.449, SRMR = 0.038. All factor loadings were significant and greater than 0.327. The correlation between the two factors was significant at 0.490. Thus, we tested a second-order model that showed the same fit as the previous model (Figure 3).

#### 3.3.4. Construct Validity

Scores of each scale of the CC-SC-CII-Al were correlated with the corresponding scales of the SC-CII-Al. Specifically, correlated coefficients of CC Self-Care Maintenance versus Self-Care Maintenance, CC Self-Care Monitoring versus Self-Care Maintenance, and Self-Care Management versus Self-Care Management scores were 0.342, 0.393, and 0.364 (*p* < 0.01), respectively.

Finally, positive correlations were found between each CC-SC-CII scale Al and PACs, the Self-affirming and Life-enriching factors (Table 3).

### 3.4. Testing the Reliability of the CC-SC-CII Albanian Version

#### 3.4.1. Internal Consistency Reliability

The internal consistency reliability computed with the composite reliability coefficients of the two CC-self-care maintenance factors, were all high (Health promoting behaviors = 0.893; Illness related behaviors = 0.890). The overall CC-self-care maintenance scale had a global reliability index for multidimensional scales of 0.837 (Table 4).

Internal consistency reliability computed with the composite reliability coefficients, of the CC-self-care monitoring scale, was also high. When Cronbach’s alpha coefficient was computed for the full 5-item scale, it yielded an adequate coefficient of 0.866.

The Internal consistency reliability of the two CC-self-care management factors was high (Autonomous behaviors = 0.825; Consulting behaviors = 0.901). The overall CC-self-care management scale had a global reliability index for multidimensional scales of 0.756.

#### 3.4.2. Stability

The reliability of the CC-SC-CII Al was assessed using test–retest reliability, where the instrument was administered again 10 days after the initial administration to a subsample of 63 dyads. The consistency between the two administrations was reflected in an intra-class correlation coefficient of 0.93.

### 3.5. Testing the Measurement Errors of the CC-SC-CII Albanian Version

The SEM for the CC-SC-CII-Al was 6.09 for CC Self-Care Maintenance, 6.26 for CC Self-Care Monitoring, and 2.19 for CC Self-Care Management. These measures were considered adequate.

The SDC was 6.84 for CC Self-Care Maintenance, 6.94 for CC Self-Care Monitoring, and 4.10 for CC Self-Care Management. These measures were also considered adequate.

## 4. Discussion

This study aims to test the measurement properties of the CC-SC-CII-A, an instrument designed to assess caregiver contribution to chronic patient self-care in a middle-income sample. The CC-SC-CII-Al demonstrated good validity and reliability properties, as shown in a previous study validating measurement properties [20]. Specifically, the CC Self-Care Maintenance scale confirmed a two-factorial structure with CC health promoting behavior and CC illness-related factors.

This result is interesting because it reveals that, despite the cultural differences among people of low–middle- and high-income countries, the construct of CC to Self-Care Maintenance among caregivers remains the same. Thus, these findings confirm that caregivers of adult patients with chronic conditions engage in self-care behaviors aimed at promoting the health of the person for whom they care (e.g., recommending the person for whom they care to manage stress) and their management of illnesses (e.g., recommending the person for whom they care to eat a special diet).

We also found an excessive residual variance between items #1 and #2, and items #5 and #6. This suggests that caregivers consider these behaviors as related. Previous studies have shown that adequate sleep is linked to lower illness rates due to its regulatory influence on immune functions [41]. Additionally, recommending the care recipient to see the healthcare provider and take prescribed medicines without missing a dose are both behaviors linked to patient’s adherence to treatment. During clinical consultations, patients frequently receive information about prescribed treatment [42], useful both for the management of new symptoms or the exacerbation of symptoms specific to the diseases the patient is suffering from. These residual covariances differed from those found in a previous validity study of the CC-SC-CII conducted on Italian caregivers [20], indicating cultural differences between countries exist [43,44,45]. The second-order hierarchical model confirmed the CC Self-Care Maintenance scale is multidimensional at the first-order factor level and unidimensional at the second-order factor level. This result proved that the construct of self-care maintenance and CC to self-care maintenance are considered as operationalized by the same self-care behaviors by both Albanian patient and caregivers.

The mono-dimensional factorial structure of the CC Self-Care Monitoring scale was also confirmed. Unlike [20], no residual covariance between item #9 and #10 was specified. Our interpretation is that caregivers in this specific population, characterized by a lower education level compared to the Italian sample [20], do not associate the behaviors of monitoring signs and symptoms with those of emotional or mental aspects as caregivers in high-income countries. Differences in healthcare systems and educational levels between Italian and Albanian caregivers may support our findings. Specifically, the lower availability and accessibility of prevention and public health programs [46], combined with a lower level of education among caregivers in Albania, may explain the reduced ability of Albanian caregivers to properly monitor signs and symptoms, including emotional aspects, compared to Italian caregivers or, more generally, those from high-income countries.

Regarding the structural validity of the Self-Care Management scale, consistent with a recent update implemented for the SC-CII and the SC-CII-Al [21], we tested a new factorial structure of the CC-SC-CII which excluded item #7 and #14 from the CC-self-care management scale. To the best of our knowledge, this is the first study that considered this update. Our analysis confirmed a two-factorial model for the CC Self-Care Management scale, composed of autonomous behaviors and consulting behavior factors. This means that caregivers contribute to managing the signs and symptoms of their care recipients by following two types of behaviors as follows: those adopted autonomously (e.g., recommending that the person for whom they care take remedies to reduce or relieve symptoms), and those performed in consultation with the healthcare provider (e.g., considering remedies that could help the person for whom they care feel better).

For this scale, we also had to specify two covariances between items #17 and #18, and #19 and #20 were observed. These correlations are not surprising, as items #17 and #18 and #19 and #20 are related to autonomous behaviors that can be associated with consulting behaviors to healthcare providers. Therapeutic and follow-up adherence represent important activities supported by caregivers of patients suffering from chronic conditions. Furthermore, calling the healthcare provider for guidance on the management of a symptom can be an opportunity for the caregiver to reflect on the effectiveness of a remedy implemented by their care recipients in the presence of symptoms.

To confirm the construct validity of the CC-SC-CII-Al, multiple hypotheses were evaluated. Positive correlations were observed between the three scales of the CC-SC-CII-Al and the corresponding scales of the SC-CII-Al, aligning with the theoretical framework that portrays self-care maintenance, monitoring, and management as interconnected dimensions of the CC process, which, in turn, influence patient self-care [11]. These relationships between patients’ self-care and caregiver contribution have been confirmed in previous studies conducted in HF and in multiple chronic diseases [47]. The study also found moderate positive correlations between the total score of PACs and each scale of the CC-SC-CII-Al, confirming that caregiver contribution to self-care is a fulfilling endeavor for family caregivers [32]. To our knowledge, no previous studies have analyzed the association between CC to patient self-care in chronic conditions and PACs, the self-affirming and life-enriching factors, a result which would need to be verified in future studies.

We found evidence of good internal consistency and reliability of the CC-SC-CII-Al, measured through indices that consider the factorial structure and the multidimensionality of the scales, the composite coefficients, and the global reliability index for multidimensional scale, respectively. To our knowledge, no previous studies have analyzed the reliability of CC-SC-CII using the composite reliability coefficients. This coefficient computed for each factor of the CC Self-Care Maintenance and CC Self-Care Management scales, together with the Cronbach’s alpha coefficient and the global reliability index for multidimensional scale, was also supportive.

Our findings demonstrate that the CC-SC-CII-Al possesses strong test–retest reliability, making it a reliable instrument for repeated use in research and clinical applications.

Finally, the SEM and SDC values for the CC-SC-CII Al subscales demonstrate that the instrument has an acceptable level of measurement error and can detect significant changes, which reinforces its utility in both research and clinical settings for evaluating and monitoring self-care behaviors.

### Limitations

Several limitations to these findings should be considered when interpreting the results. First, the use of a convenient balanced sample from a multicenter enrollment. Second, the overrepresentation of caregivers with low education in our sample. Unexpectedly, about 96% of the caregivers had less than 8 years of education, which could be explained by the limited opportunities for higher education available to Albanian citizens during the communist period in the 1980s and 1990s. Another limitation is that caregivers were enrolled from only one LMIC. Further studies on measurement properties are recommended to generalize the properties to other LMICs. Finally, we excluded caregivers of patients with dementia and cancer. For this reason, we cannot assume the validity and reliability of the CC-SC-CII-Al in caregivers of patients with dementia and/or cancer.

## 5. Conclusions

The CC to self-care is an essential element in the care of chronic illness. The CC-SC-CII-Al demonstrated strong measurement properties, confirming it is a valid and reliable instrument for assessing CC to patient self-care in a MIC context, such as Albania. Furthermore, our results indicate that, despite differences between LMICs and high-income countries, the construct of caregiver contributions to self-care remains consistent. The CC-SC-CII-Al could be valuable for both clinical practice and research. This theory-based instrument for caregivers enables clinicians to assess caregiver contribution, providing a comprehensive overview of chronic condition self-care and shared management.

## Figures and Tables

**Table 1:** Socio-demographic and clinical characteristics of caregivers (N = 250) and their patients (N = 250).

Variable	N (%)
Gender	
Female	166 (66.40)
Male	84 (33.60)
Marital Status	
Married	207 (82.80)
Single	35 (14.0)
Separated/Divorced/Widowed	8 (3.20)
Education level (years)	
≤8	242 (96.80)
>8	89 (3.20)
Income	
Enough to live	200 (80.0)
More than necessary to live	34 (12.80)
Lower than necessary to live	18 (7.20)
Living with other people	
No	2 (0.80)
1	77 (30.80)
2	58 (23.20)
≥3	113 (45.20)
Living with patient	
Yes	174 (69.60)
No	76 (30.40)
Relationship patient–caregiver	
Sons/Daughter	96 (38.40)
Husband/Wife	78 (31.20)
Son/Daughter-in-law	40 (16.0)
Nephew/Niece	23 (9.20)
Brother/Sister	5 (2.0)
Other	8 (3.20)
Patient’s chronic conditions	
Hypertension	219 (87.6)
Diabetes mellitus	185 (74.0)
Heart failure	112 (44.8)
COPD	32 (12.8)
Kidney disease	20 (8.0)
Arthritis	20 (8.0)
Other	28 (11.2)
	(Mean ± DS)
Age	49.91 (15.59)
Caregiving hours per week	
0–10	56 (22.40)
11–20	95 (38.0)
21–30	78 (31.20)
>30	21 (8.40)
Number of patients with chronic conditions	2.49 (0.70)

Legend. COPD, chronic obstructive pulmonary conditions; DM, diabetes mellitus; NYHA, New York Heart Association; GOLD, Global Initiative for Chronic Obstructive Lung Conditions; HF, heart failure; mMRC, modified British Medical Research Council questionnaire; SD, standard deviation.

**Table 2:** Item description of Albanian version of the Caregiver Contribution to Self-Care of Chronic Illness Inventory (n = 250 caregiver).

Items	M	SD	Skewness	Kurtosis
*How often do you recommend that the person you care for do the following things?*				
1. Get enough sleep	4.43	0.80	−1.45	2.15
2. Try to avoid getting sick (e.g., flu shot, wash their hands)	4.55	0.73	−1.71	2.90
3. Do physical activity (e.g., take a brisk walk, use the stairs)	3.99	1.05	−0.67	−0.56
4. Eat a special diet	4.14	0.93	−0.66	−0.61
5. See their healthcare provider for routine healthcare	4.57	0.71	−1.69	2.41
6. Take prescribed medicines without missing a dose	4.74	0.58	−2.78	9.71
8. Manage stress	4.02	1.05	−0.65	−0.73
*How often do you do the following things?*				
9. Monitor the condition of the person for whom you care you care?	4.50	0.67	−0.99	−0.23
10. Pay attention to changes in how the person for whom you care feels?	4.31	0.82	−1.07	0.70
11. Monitor for medication side-effects of the person for whom you care?	4.13	0.88	−0.57	−0.59
12. Monitor whether the person for whom you care tires more than usual doing normal activities?	4.15	0.89	−0.65	−0.63
13. Monitor for symptoms of the person for whom you care?	4.24	0.84	−0.78	−0.35
15. When the person for whom you care has symptoms, how likely are you to recommend or actually change what he/she eats or drinks to make the symptom decrease or go away?	4.13	0.85	−0.64	−0.17
16. When the person for whom you care has symptoms, how likely are you to recommend or actually change his/her activity level (e.g., slow down, rest)?	4.26	0.80	−0.71	−0.50
17. When the person for whom you care has symptoms, how likely are you to recommend he/she take medicines to make the symptoms decrease or go away?	4.43	0.76	−1.13	0.78
18. When the person for whom you care has symptoms, how likely are you to recommend he/she tell his/her healthcare provider about the symptoms at the next office visit?	4.53	0.71	−1.38	1.25
19. When the person for whom you care has symptoms, how likely are you to recommend he/she call his/her healthcare provider for guidance?	4.18	0.97	−0.94	0.01
20. Think of a remedy you tried the last time the patient for whom you care had symptoms. Did the remedy make the person you care for feel better?	3.51	1.16	−0.70	0.56

Legend. SD, standard deviation; M, mean. Items #7 (“avoid tobacco smoke”) and #14 (how quickly did you recognize the symptom of the illness patient is suffering from) were excluded from all analyses, consistent with previous studies conducted on the SC-CII by the scale’s authors.

**Table 3:** Bivariate correlation of construct validity of Caregiver Contribution to patient self-care.

Variable	1	2	3	4	5	6
1. CC Self-Care Maintenance	-					
2. CC Self-Care Monitoring	0.584					
3. CC Self-Care Management	0.600	0.767				
4. Self-Care Maintenance	0.342	0.250	0.191			
5. Self-Care Monitoring	0.273	0.393	0.233	0.600		
6. Self-Care Management	0.361	0.396	0.364	0.561	0.631	
7. PACs	0.231	0.289	0.249	0.326	0.128 *	0.233

Legend. CC, caregiver contribution; PACs, positive aspect of caregiving scale. Note. All correlations are significant at the 0.01 level (two-tailed) except those marked with *. * The correlation is significant at the 0.05 level (two-tailed).

**Table 4:** Internal consistency reliability of single factors and overall CC-SCCII-Al scales.

Variable	Single Factor Reliability	Multidimensional Model-Based Reliability	Cronbach’s Alpha
CC Self-Care Maintenance scale		0.837	0.833
Health promoting behaviors factor	0.893		
Illness-related behavior factor	0.890		
CC Self-Care Monitoring scale	-	-	0.866
CC Self-Care Management scale		0.756	0.728
Autonomous behavior factor	0.825		
Consulting behavior factor	0.901		

## Data Availability

Dataset is available upon request from the authors.

## Public Involvement Statement

No public involvement in any aspect of this research is reported.

## Guidelines and Standards Statement

This manuscript was drafted against the STROBE Statement for cross-sectional studies.
